# The effects of excess weight on cardiac strain and steatosis in adults and children

**DOI:** 10.1186/1532-429X-15-S1-O30

**Published:** 2013-01-30

**Authors:** Rajarshi Banerjee, Belen Rial, Joseph Suttie, Ntobeko Ntusi, Adam J Lewandowski, Oliver J Rider, Matthew D Robson, Jurgen E Schneider, Paul Leeson, Stefan Neubauer

**Affiliations:** 1 Oxford Centre for Clinical Magnetic Resonance Research, University of Oxford, Oxford, UK; 2 Department of Cardiovascular Medicine, University of Oxford, Oxford, UK

## Background

Excess body fat is a known risk factor for cardiovascular mortality and morbidity. However, obesity, hypertension, hyperlipidaemia and insulin resistance are closely interlinked, both in their causes (eg sedentary lifestyle, social class, diet & age) and effects (endothelial function, increased left ventricular mass, increased myocardial fatty acid uptake). Thus it has been difficult to determine the exact effects of excess weight alone on myocardial function and metabolism. Myocardial triglyceride deposition and impaired strain have been clearly shown in adult diabetic cardiomyopathy. Recently, similar findings have been demonstrated in obese non-diabetic women. We set out to see if obesity alone, in the absence of other components of the Metabolic Syndrome, was also associated with cardiac steatosis and/or impaired peak circumferential strain in adults of both sexes and in adolescent boys.

## Methods

We recruited 100 adults (44 lean, 28 overweight & 28 obese) and 22 boys aged 10-15 years (11 lean & 11 obese) by open poster advertisement. Exclusion criteria were heart failure, valvular disease, the use of any cardiovascular medications and any classical risk factors (hypertension, hyperlipidaemia, diabetes mellitus, smoking and obstructive sleep apnoea). Body mass index (BMI) and waist circumference were measured in all subjects. Left ventricular (LV) mass, volumes, function and peak circumferential strain were measured with 3T cardiac MR imaging and tagging. Myocardial triglyceride content (MTGC) was determined by ECG-gated localised proton spectroscopy to calculate the lipid : water ratio in the interventricular septum. Image and spectroscopy data were anonymised prior to analysis.

## Results

**Figure 1:**
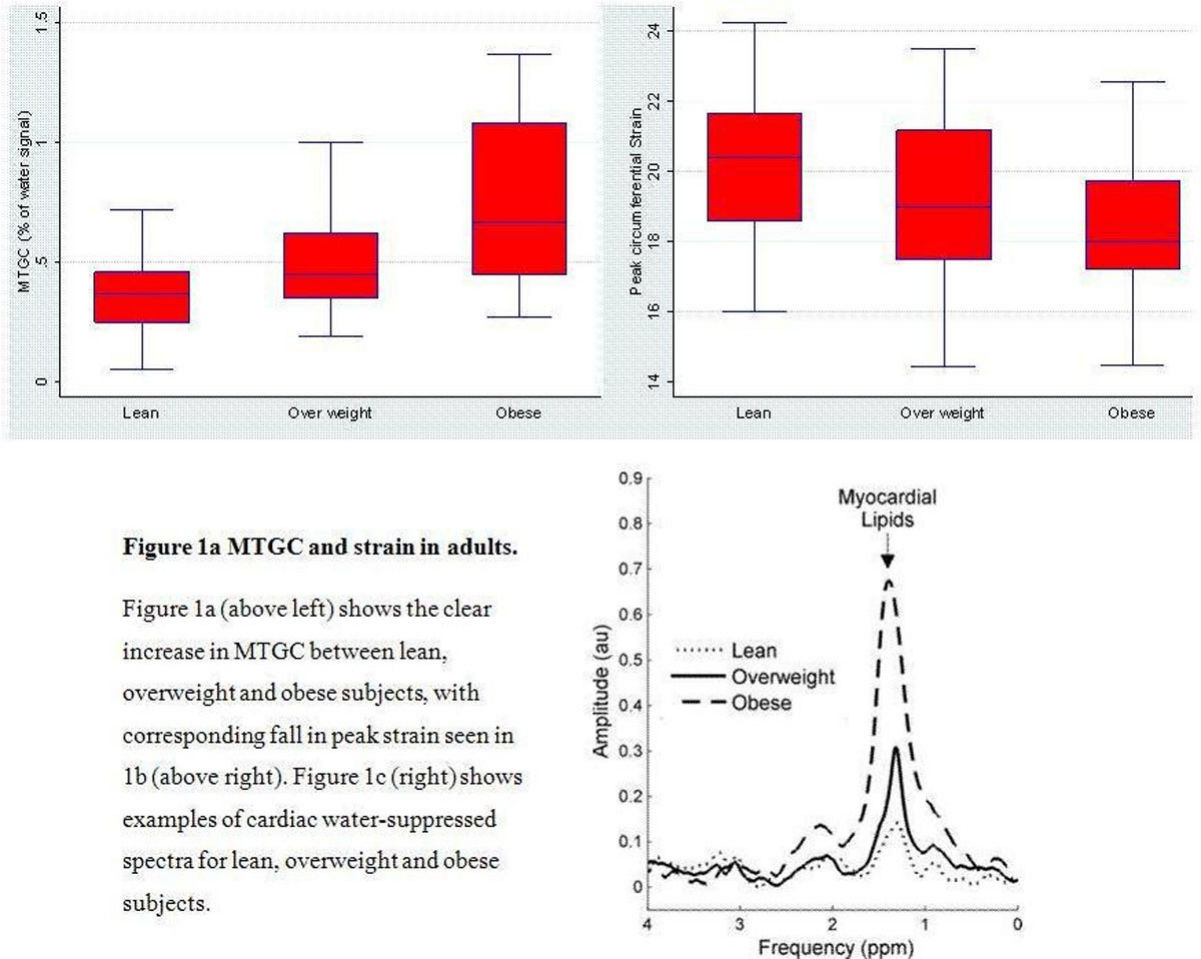


There was a clear increase in myocardial lipid content in overweight and obese adults compared to the lean control group (Figure 1). Body mass index and waist circumference were associated with a linear increase in MTGC (a 10kg/m^2^ increase in BMI was associated with a 48% increase in MTGC, p < 0.05), but gender had no impact. There was also an 11% reduction in the peak circumferential strain of obese adults compared to their lean peers (median -18.0 v -21.4, p = 0.01), despite similar LV ejection fractions. LV mass was 16% higher in overweight and 20% higher in obese adults (p <0.01).

**Figure 2:**
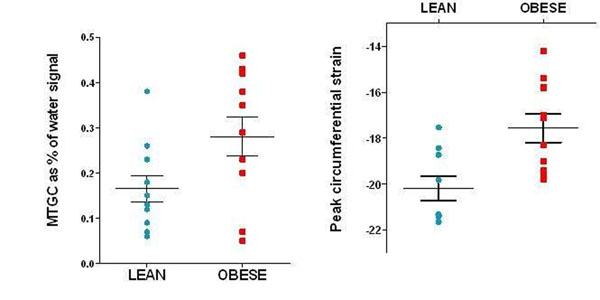
Obese boys had significantly higher MTGC (left) and impaired strain (right).

In the adolescent group, obese boys had higher mean myocardial triglyceride deposition (0.29% v 0.16%, p = 0.04) and impaired strain (-17.5 v -20.2, p < 0.01), but with no significant difference in LV mass, ejection fraction, end-diastolic volume, glucose or cholesterol (Figure 2).

## Conclusions

Obesity in the absence of diabetes or Metabolic Syndrome is associated with significant myocardial triglyceride deposition and impaired strain in adults and children. In paediatric obesity, this data suggests that myocardial steatosis and change in strain occur before any hypertrophy. The assessment of strain and MTGC may help in the diagnosis and monitoring of obesity-related heart disease, especially in children.

## Funding

British Heart Foundation.

